# Association of hyponatremia and risk of in-hospital mortality in patients with acute stroke: a systematic review and meta-analysis

**DOI:** 10.3389/fstro.2026.1816483

**Published:** 2026-05-15

**Authors:** Prachi Sharma, Anuradha Pawar, Manabesh Nath, Deepti Vibha, Pradeep Kumar

**Affiliations:** All India Institute of Medical Sciences, New Delhi, India

**Keywords:** acute stroke, hyponatremia, in-hospital mortality, meta-analysis, prognostic marker, serum sodium

## Abstract

**Objective:**

Hyponatremia is among the most common electrolyte disturbances in acute stroke and has been increasingly recognized as a potential predictor of early complications. We aimed to evaluate whether hyponatremia on admission is associated with increased in-hospital mortality in patients with acute ischemic stroke (IS) and hemorrhagic stroke (HS).

**Methods:**

We conducted a comprehensive systematic search of PubMed, Scopus, and Embase to identify relevant studies published between 1st January 2000 and 30th October 2025. Eligible studies included patients with acute stroke, reported admission serum sodium levels, and compared outcomes between hyponatremic and normonatremic groups. Pooled risk ratios (RRs) with 95% confidence intervals (CIs) were estimated using a random-effects model. Subgroup analyses were performed based on stroke type.

**Results:**

Seventeen studies involving patients assessed within 24–72 h of stroke onset were included. Hyponatremia on admission was significantly associated with higher in-hospital mortality (RR 1.41, 95% CI 1.14–1.74). The association remained significant in ischemic stroke (RR 1.29, 95% CI 1.16–1.42) and in studies including mixed IS and HS populations (RR 1.59, 95% CI 1.19–2.12). No significant association was observed in hemorrhagic stroke alone (RR 1.47, 95% CI 0.70–3.10). Between-study heterogeneity was moderate (*I*^2^ = 58.9%, *p* < 0.001). Funnel plot assessment suggested no significant publication bias.

**Conclusions:**

Hyponatremia on admission is associated with increased in-hospital mortality in acute stroke, particularly in ischemic stroke. Early identification and management of hyponatremia may have prognostic and therapeutic relevance in acute stroke care.

**Systematic Review Registration:**

PROSPERO: CRD420251110001.

## Introduction

Acute stroke disrupts cerebral homeostasis through ischemic or hemorrhagic injury, leading to rapid cellular and microvascular dysfunction (Salaudeen et al., 2024). Early alterations in sodium-dependent mechanisms contribute to cerebral edema, impaired perfusion, and neurological deterioration, thereby linking systemic sodium balance with brain water regulation and neuronal survival (Gankam Kengne, 2023). Serum sodium, a key determinant of extracellular osmolality, plays a central role in maintaining cerebral cell volume (Gankam Kengne and Decaux, 2017). Hyponatremia in acute stroke may arise from multiple mechanisms, including syndrome of inappropriate antidiuresis, cerebral salt wasting, and treatment-related factors, and may both reflect neuroendocrine stress and exacerbate ongoing cerebral injury, suggesting a bidirectional relationship between dysnatremia and brain injury (Cui et al., 2019; Liamis et al., 2019).

Importantly, the timing of serum sodium assessment is a critical methodological consideration. Sodium levels measured beyond the initial 24–72 h may be influenced by iatrogenic factors such as fluid therapy or underlying comorbidities, rather than the direct effects of the acute cerebrovascular event (Yuen et al., 2022). Variability in the timing of sodium measurement across studies may therefore contribute to heterogeneity and inconsistent findings regarding its prognostic significance.

Despite strong biological plausibility, the association between hyponatremia on admission and clinical outcomes in acute stroke remains uncertain. Several observational studies have reported links with adverse outcomes, including early neurological deterioration and mortality; however, results have been inconsistent, likely due to differences in study populations, definitions, and methodological approaches (Huang et al., 2012; Ha and Ryu, 2021; Qian et al., 2025). Therefore, we conducted a systematic review and meta-analysis to evaluate the association between hyponatremia on admission and in-hospital mortality in patients with acute stroke, with the aim of clarifying its prognostic relevance and informing early risk stratification.

## Methodology

### Protocol registration

This systematic review and meta-analysis was designed and reported in accordance with the Preferred Reporting Items for Systematic Reviews and Meta-Analyses (PRISMA) recommendations (Moher et al., 2015). The study protocol was registered prospectively with the International Prospective Register of Systematic Reviews (PROSPERO; Registration number: CRD420251110001) before commencement of the review, to enhance transparency and ensure methodological rigor.

### Eligibility criteria

- Study framework: study selection was based on the Population, Intervention/Exposure, Comparator, Outcomes, Study design (PICOS) framework.
- Population: adult patients aged ≥18 years presenting with:

- Acute ischemic stroke
- Hemorrhagic stroke
- Mixed stroke cohorts

- Exposure: hyponatremia at hospital admission, defined according to the prespecified serum sodium thresholds reported in individual studies.
- Comparator: patients with normonatremia at hospital admission.
- Outcome: in-hospital mortality.
- Study design: observational studies, including:

- Cross-sectional studies (CSS)
- Prospective cohort studies (PCS)
- Retrospective cohort studies (RCS)
- Case–control studies

### Exclusion criteria

- Studies without extractable data on admission serum sodium levels.
- Studies that did not report in-hospital mortality or provided insufficient data to calculate effect estimates.
- For overlapping datasets, only the most comprehensive or methodologically robust study was included.

### Search strategy

A comprehensive literature search was conducted in PubMed, Embase, and Scopus to identify relevant studies published between 1st January 2000 and 30th October 2025. The search strategy combined free-text keywords and controlled vocabulary terms related to hyponatremia and acute stroke, including “hyponatremia,” “sodium,” “dysnatremia,” “ischemic stroke,” “Intracerebral hemorrhage,” and “acute stroke.” Boolean operators and database-specific search filters were applied as appropriate. Additionally, the reference lists of included studies and relevant review articles were manually screened to identify further eligible publications. The detailed search strategy is provided in Supplementary Table S1. No language restrictions were applied.

### Data extraction

Two reviewers (PS and AP) independently screened titles, abstracts, and full-text articles to determine study eligibility. Data were extracted using a predefined, standardized extraction form and included information on study characteristics, patient demographics, serum sodium definitions, stroke subtype, timing of sodium assessment, and in-hospital mortality outcomes. Discrepancies between reviewers were resolved through discussion and consensus, with adjudication by the corresponding author (PK) when required.

### Statistical analysis

Risk ratios (RR) with corresponding 95% confidence intervals (CIs) were calculated to evaluate the association between hyponatremia at hospital admission and in-hospital mortality. Adjusted effect estimates were preferentially used when available. Pooled estimates were derived using a random-effects model using the DerSimonian–Laird method to account for anticipated clinical and methodological heterogeneity. Statistical heterogeneity was assessed using the Cochran *Q* test and quantified using the *I*^2^ statistic (Higgins et al., 2003). Pre-specified subgroup analyses were conducted based on stroke subtype (ischemic, hemorrhagic, or mixed), timing of serum sodium assessment (at admission vs. within 24–48 h), and ethnicity of the study population. Publication bias was evaluated through visual inspection of funnel plots and quantitatively using Egger's regression test. All statistical analyses were performed using STATA version 16.1 (StataCorp, College Station, TX, USA), with a two-sided *p* value <0.05 considered statistically significant.

## Results

### Study selection

**Figure 1:**
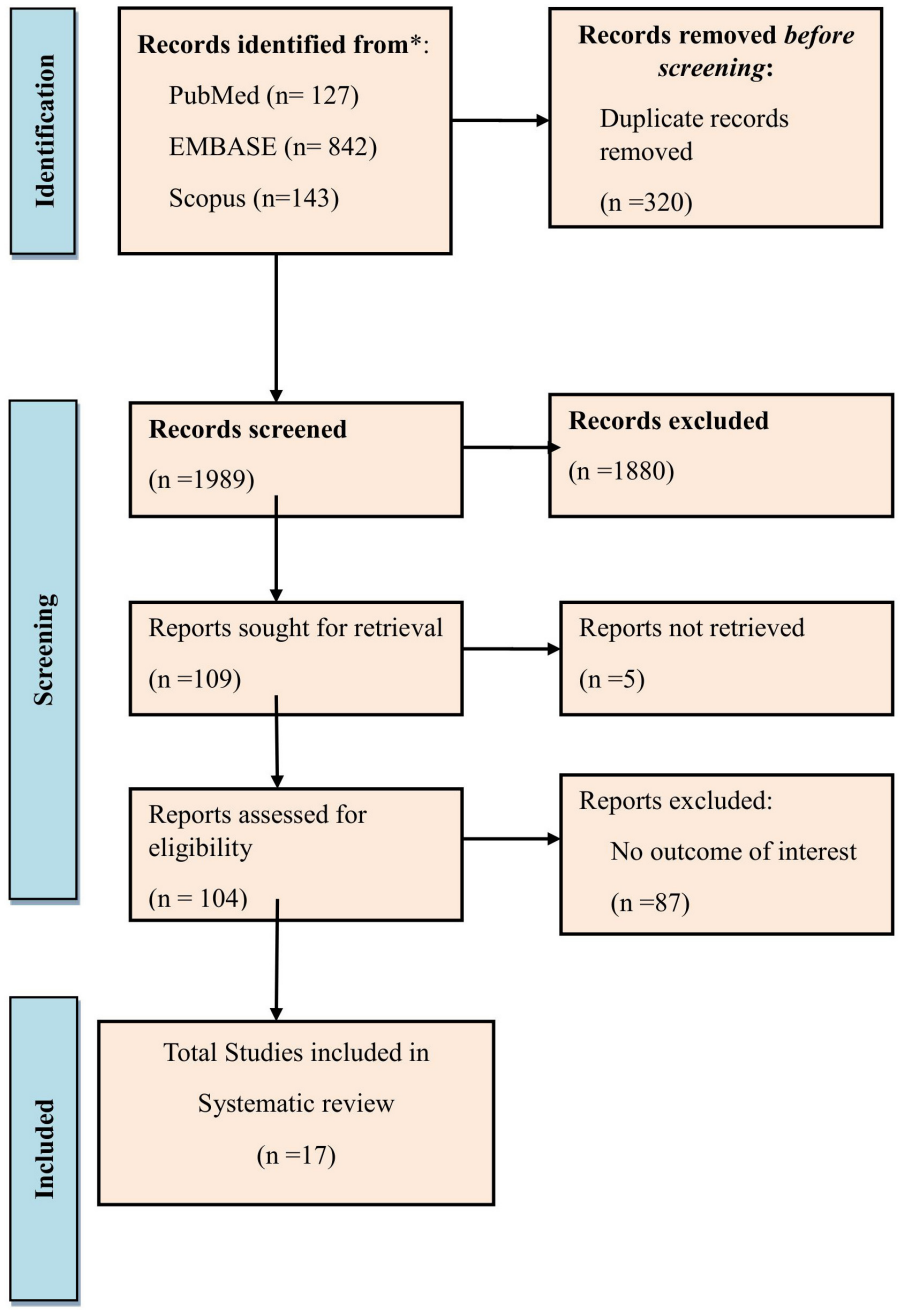
PRISMA 2020 flow diagram for the selection of studies.

The systematic literature search yielded 2,309 records, of which 1,880 unique citations remained after duplicate removal. After screening titles and abstracts, 104 articles were selected for full-text assessment. Ultimately, 17 studies fulfilled all predefined eligibility criteria and were included in the final analysis. The primary reasons for exclusion at the full-text stage included the absence of extractable data on in-hospital mortality, lack of an appropriate normonatremic comparator group, and outcomes that were not relevant to the objectives of the review. The study selection process is depicted in the PRISMA flow diagram (Figure 1).

### Study characteristics

The 17 included studies were published between 2014 and 2025 and collectively evaluated patients with acute stroke across diverse geographic regions. Most studies were conducted in Asia (India, Pakistan, Bangladesh, Sri Lanka, Nepal and Iran), with additional contributions from Europe, North America, and Australia. Study designs included retrospective cohort (RCS), prospective cohort (PCS), cross-sectional (CSS), reflecting a mix of observational methodologies (Table 1). All studies focused on the acute stage of stroke, with diagnosis generally confirmed by CT and/or MRI, and in some cases supported by clinical criteria or standardized classifications such as AHA/ASA or TOAST. Admission timing varied, although many studies measured serum sodium within the first 24–48 h of hospitalization.

**Table 1:** Characteristics of included studies for investigating for the association of hyponatremia and risk of in-hospital mortality in patients with acute stroke.

S. No	Author year	Country	Ethnicity	Study design	Study period	Stage ofstroke	Diagnosticcriteria	Time ofadmission	Total samplesize	M/F	Age (Mean ± SD)	Cut-off value of Na (mmol/L)	Outcome investigated
1	Kuramatsu et al. (2014)	Germany	Caucasian	RCS	2006–2010	Acute	CT/MRI	Within 24 h	422	NR	69.7 ± 12.0	<135	In hospital, 90 day, and 1 year mortality; FO
2	Rodrigues et al. (2014)	USA	Caucasian	RCS	2004–2014	Acute	Clinical + CT/MRI	Within 24–72 h	3,541	1,725/1,816	71	<135	In hospital, mortality at 90 d, mortality at 1 year
3	Gray et al. (2014)	USA	Caucasian	RCS	Jan 2006–Jul 2009	Acute	Clinical + CT/MRI	Within 48 h	99	61/38	58.8	<135	Hospital LOS, ICU LOS, In hospital mortality
4	Adekunle-Olarinde et al. (2017)	UK	Caucasian	RCS	Jan 2003–May 2015	Acute	Clinical + SOAR Score	Within 24 h	8,493	4,025/4,468	77.7 ± 11.6	<135	Inpatient and 7 d mortality
5	Kalita et al. (2017)	India	Asian	PCS	Jan 2014–Jan 2016	Acute	CT/MRI	Within 48 h	100	69/31	62 (18–90)	<135	Disabilty, In-hospital mortality
6	Shah et al. (2019)	Karachi	Asian	PCS	Jul 2018–Dec 2018	Acute	Clinical + CT/MRI	Within 24–72 h	234	182/52	59.14 ± 9.05	<135	Recovery, disability, In hospital mortality
7	Hakim et al. (2019)	Bangladesh	Asian	CSS	Feb 2017–Nov 2017	Acute	Clinical + CT/MRI	Within 24–72 h	205	124/81	60 ± 13.1	<135	Hospital LOS, In hospital mortality
8	Quinn et al. (2020)	Australia	Caucasian	PCS	Jan 2015–May 2019	Acute	Clinical + CT	Within 24 h	200	56/144	57 ± 12	<135	ICU mortality, Hospital mortality, FU
9	Saadat et al. (2022)	Iran	Asian	CSS	Jan 2021–Jun 2021	Acute	AHA/ASA +TOAST classification, CT/MRI	Within 24 h	125	55/70	64.8 ± 8.3	<135	Mortality, disability, severity
10	Padhy et al. (2023)	India	Asian	CSS	Mar 2021–May 2021	Acute	Clinical + CT/MRI	Within 24 h	100	46/54	64.2	<135	In hospital mortality, 60 d mortality
11	Khan et al. (2023)	Pakistan	Asian	CSS	Sep 2022–Feb 2023	Acute	AHA/ASA	Within 24 h	289	162/127	61 ± 7.93	<135	Severity, In-hospital mortality
12	Ranasinghe et al. (2023)	Sri Lanka	Asian	PCS	June 2019–Feb 2021	Acute	Clinical + CT	Within 24 h	246	120/126	68.14 ± 12.71	<131	In hospital, 70 d mortality
13	Ezhumalai et al. (2023)	India	Asian	PCS	Oct 2021–Dec 2021	Acute	Clinical + CT/MRI	Within 24–72 h	77	56/21	56	<135	Mortality
14	Pandya et al. (2024)	India	Asian	CSS	Jan 2023–June 2023	Acute	CT/MRI	Within 48 h	210	155/55	58.5 ± 11.0	<135	Mortality
15	Malhotra et al. (2024)	India	Asian	PCS	Mar 2021–Jul 2022	Acute	Clinical	Within 24–72 h	110	70/40	>60	<135	Severity; Mortality
16	Sah et al. (2024)	Nepal	Asian	CSS	Jan 2023–Jan 2024	Acute	CT/MRI	Within 24 h	100	63/37	64	<135	Severity; Mortality
17	Naidu and Kumari (2025)	India	Asian	RCS	Dec 2023–Dec 2024	Acute	CT/MRI	Within 24–72 h	60	40/20	NR	<135	Mortality

SOAR Score, Stroke subtype Oxfordshire Community Stroke Project Classification Age Rankin(pre-stroke modified) scale; AHA/ASA, American Heart Association/American Stroke Association; CSS, cross-sectional study; PCS, prospective cohort study; RCS, retrospective cohort study; CT, computed tomography; MRI, magnetic resonance imaging; Na, sodium; ICU, intensive care unit; LOS, length of stay; FO, functional outcome; FU, follow-up; NR, not reported; M/F, Male/Female; SD, standard deviation; d, days; h, hour.

The total sample sizes ranged widely, from 60 to 8,493 participants, with most cohorts including both men and women. Hyponatremia was most commonly defined using a serum sodium cutoff of <135 mmol/L. Outcomes assessed across studies primarily included in-hospital mortality, with several studies also reporting short-term mortality (7–90 days), functional outcomes, disability, stroke severity, or length of hospital stay. Despite methodological and clinical heterogeneity, the included studies were broadly comparable in their focus on the prognostic role of hyponatremia on admission in acute stroke.

### Main results

**Figure 2:**
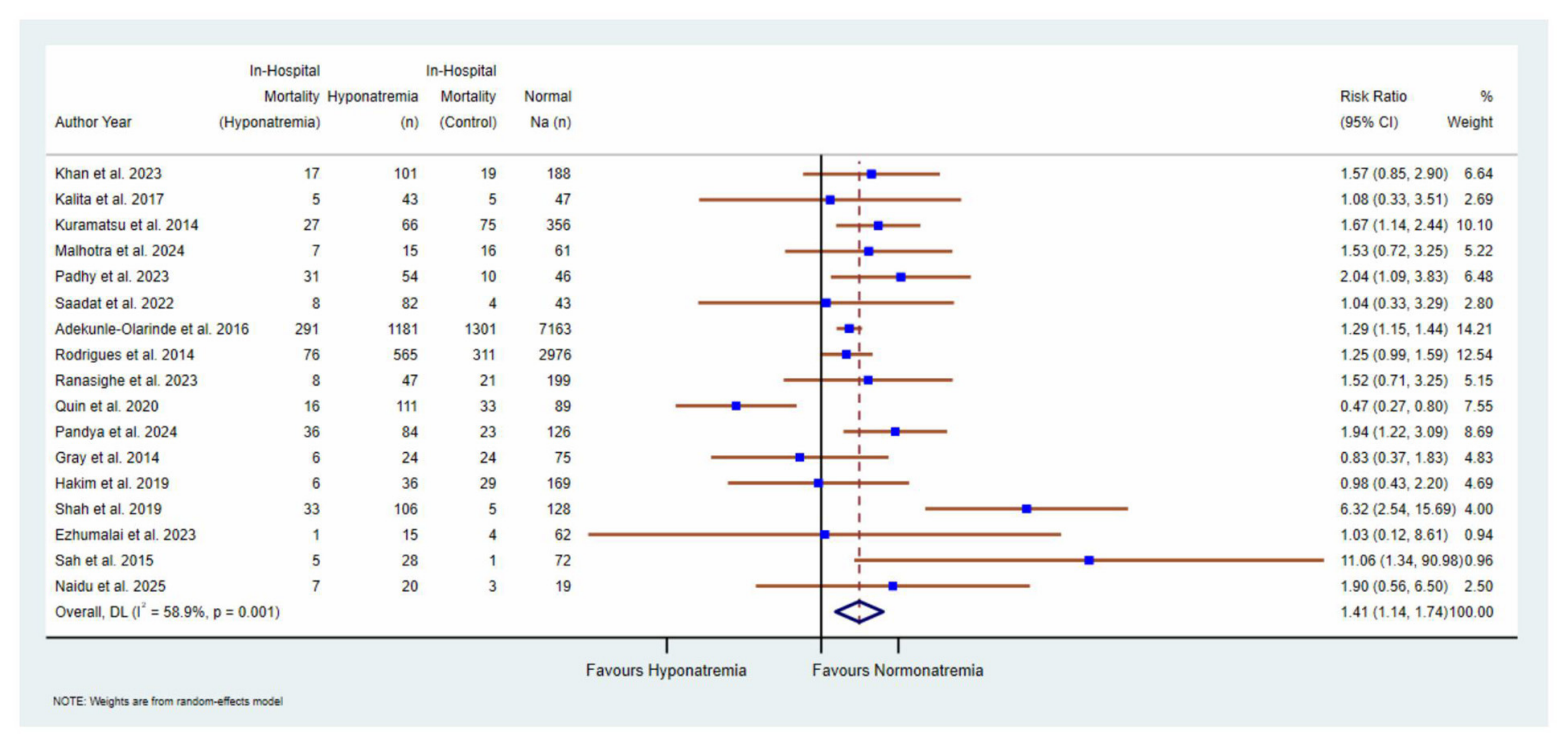
Forest plot demonstrating the association between hyponatremia and in-hospital mortality stratified by stroke subtype.

Overall, hyponatremia on admission was significantly associated with an increased risk of in-hospital mortality in patients with acute stroke (RR = 1.41; 95% CI, 1.14–1.74), with moderate heterogeneity across studies (*I*^2^ = 58.9%; Figure 2).

In subgroup analyses by stroke subtype, hyponatremia was associated with higher mortality in ischemic stroke (RR = 1.29; 95% CI, 1.16–1.42; *I*^2^ = 0.0%) and in mixed ischemic and hemorrhagic stroke cohorts (RR = 1.59; 95% CI, 1.19–2.12; *I*^2^ = 0.0%). In hemorrhagic stroke, the association was directionally similar but highly heterogeneous and did not reach statistical significance (RR = 1.47; 95% CI, 0.70–3.0; *I*^2^ = 86.7%).

Ethnicity-based subgroup analysis demonstrated a significantly higher mortality risk associated with hyponatremia among Asian populations (RR = 1.78; 95% CI, 1.34–2.38; *I*^2^ = 26.7%). In contrast, no significant association was observed in predominantly Caucasian populations (RR = 1.11; 95% CI, 0.84–1.48), with considerable heterogeneity (*I*^2^ = 75.3%).

Subgroup analysis based on the timing of serum sodium assessment showed an increased mortality risk when sodium was measured within 48 h (RR = 1.33; 95% CI, 0.74–2.41; *I*^2 =^ 45.3%), although these associations were not statistically significant. Studies reporting sodium assessment within 24 h demonstrated a modest, non-significant increase in mortality (RR = 1.34; 95% CI, 0.96–1.86; *I*^2^ = 68.3%). A significant association was observed in studies reporting sodium assessment within 24–72 h (RR = 1.68; 95% CI, 1.00–2.81; *I*^2^ = 59.9%; Supplementary Table S2).

When studies were stratified by the timing of sodium measurement (within 24, 48, and 24–72 h), the direction and magnitude of the association between hyponatremia and in-hospital mortality remained consistent across subgroups. There was no significant interaction between timing subgroups (*p* = 0.750), indicating that the prognostic impact of hyponatremia was not materially influenced by when serum sodium was measured during hospitalization.

### Publication bias

**Figure 3:**
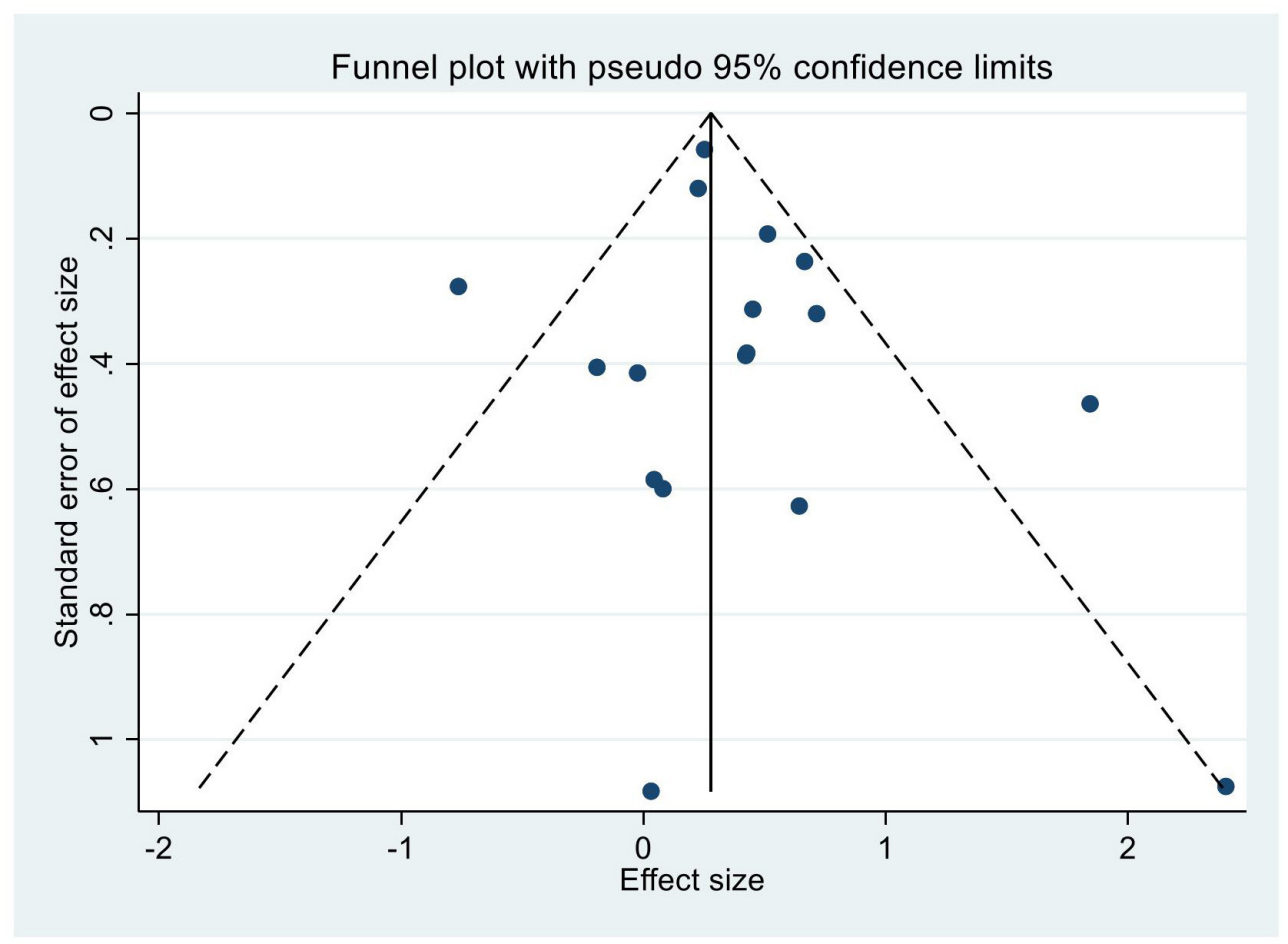
Funnel plot for the association between hyponatremia and in-hospital mortality.

Visual inspection of the funnel plot showed no substantial asymmetry, indicating a low risk of publication bias (*p* = 0.40; Figure 3). The majority of studies were symmetrically distributed around the pooled effect estimate with a consistent direction of association, supporting the robustness of the findings.

## Discussion

This systematic review and meta-analysis demonstrates that hyponatremia on admission is associated with a significantly increased risk of in-hospital mortality in patients with acute stroke. Across 17 studies with diverse geographic representation and study designs, hyponatremia conferred an approximately 40% higher mortality risk, with no evidence of publication bias, supporting the robustness of the association.

The observed association is biologically plausible. In acute stroke, hyponatremia often reflects neuroendocrine dysregulation, including syndrome of inappropriate antidiuretic hormone secretion and cerebral salt wasting, both of which are linked to greater cerebral injury and raised intracranial pressure (Pandya et al., 2024). Reduced serum sodium promotes osmotic cerebral edema, impairs neuronal function, and disrupts cerebral perfusion, thereby increasing the risk of early neurological deterioration and death (John et al., 2025). Even mild hyponatremia has been shown to adversely affect neuronal excitability and cerebral metabolism, compounding ischemic injury (Gankam Kengne and Decaux, 2017).

Existing literature consistently supports the prognostic relevance of hyponatremia in acute neurological illnesses, including stroke. Previous observational studies have shown that hyponatremia is independently associated with increased mortality, longer hospital stay, and poorer functional outcomes in stroke patients (Eldhose et al., 2025; Soiza et al., 2015). Mechanistically, hyponatremia reflects central nervous system–mediated dysregulation of water and sodium homeostasis, commonly driven by syndrome of inappropriate antidiuretic hormone secretion or cerebral salt wasting following acute brain injury (Cui et al., 2019). These disturbances exacerbate cerebral edema, compromise cerebral perfusion, and increase intracranial pressure, thereby amplifying secondary brain injury (Gankam Kengne and Decaux, 2017). Moreover, hyponatremia has been linked to heightened inflammatory responses, autonomic dysfunction, and cardiovascular instability, all of which contribute to early clinical deterioration (Park and Shin, 2013). The consistent association observed across diverse populations and study designs in the present review reinforces prior evidence and underscores hyponatremia as not merely a biochemical abnormality, but a pathophysiological meaningful marker of disease severity and early physiological decompensating in acute stroke.

Subgroup analyses showed a consistent association in ischemic and mixed stroke populations, with low heterogeneity, whereas findings in hemorrhagic stroke were less consistent, likely reflecting differences in underlying pathophysiology, fluid management practices, and smaller sample sizes. The stronger association observed in Asian populations may relate to population-specific factors such as baseline sodium levels, dietary patterns, stroke characteristics, or healthcare delivery systems. Variability in diagnostic criteria across the included studies represents an important source of heterogeneity. Differences in the definition of stroke, methods of confirmation (clinical vs. imaging-based), and thresholds used for defining hyponatremia may have influenced patient classification and outcome assessment.

Notably, the prognostic impact of hyponatremia was not materially influenced by the timing of sodium measurement or study quality, indicating that hyponatremia at any point during early hospitalization carries prognostic significance. Beyond direct neurological effects, hyponatremia may also serve as an integrative marker of systemic illness severity, capturing inflammatory, metabolic, and cardiovascular stress responses that contribute to adverse outcomes (Podestà et al., 2015). Collectively, these findings support serum sodium as a simple, readily available, and clinically meaningful prognostic marker in acute stroke care.

### Limitations

Several limitations merit consideration: (1) most included studies were observational, limiting causal inference and allowing for residual confounding; (2) heterogeneity in the definition and timing of hyponatremia assessment and adjustment for stroke severity may have influenced effect estimates; (3) limited data on the etiology, severity, duration, and correction of hyponatremia precluded dose–response and treatment-effect analyses; (4) outcomes were largely restricted to in-hospital mortality, with insufficient data on long-term functional and survival outcomes; (5) an important limitation of the present analysis is the lack of detailed and standardized reporting on the management of hyponatremia across included studies. Most studies did not provide sufficient information regarding specific therapeutic interventions (e.g., use of hypertonic saline), timing or protocols for sodium correction, or the direct impact of such treatments on clinical outcomes, including in-hospital mortality. Consequently, a quantitative synthesis evaluating the effect of hyponatremia correction on outcomes was not feasible, and (6) the predominance of studies from Asian populations may limit the generalizability of the findings to other regions.

## Conclusion

In patients with acute stroke, hyponatremia on admission is independently associated with a significantly increased risk of in-hospital mortality. This association is consistent across stroke subtypes and timing of serum sodium assessment, and is not driven by any single study. These findings highlight hyponatremia as an important early and readily available prognostic marker in acute stroke care, underscoring the need for prompt recognition and appropriate management to potentially improve short-term outcomes. Further prospective studies are warranted to elucidate causal mechanisms and to determine whether targeted correction of hyponatremia can favorably influence clinical outcomes in stroke patients.

## Data Availability

The original contributions presented in the study are included in the article/Supplementary material, further inquiries can be directed to the corresponding author.

## References

[B1] Adekunle-Olarinde I. R. McCall S. J. Barlas R. S. Wood A. D. Clark A. B. Bettencourt-Silva J. H. . (2017). Addition of sodium criterion to SOAR stroke score. Acta Neurol. Scand. 135, 553–559. doi: 10.1111/ane.12634 27397108

[B2] Cui H. He G. Yang S. Lv Y. Jiang Z. Gang X. (2019). Inappropriate antidiuretic hormone secretion and cerebral salt-wasting syndromes in neurological patients. Front Neurosci. 13:1170. doi: 10.3389/fnins.2019.01170 31780881 PMC6857451

[B3] Eldhose R. Gopinath K. G. Thomas J. C. (2025). Electrolyte imbalance in elderly stroke patients: a retrospective analysis from a tertiary center. J. Indian Acad. Geriatr. 21:105. doi: 10.4103/jiag.jiag_3_25

[B4] Ezhumalai A. Sridevi S. N. Kannan N. Kishore R. (2023). A prospective study of evaluation and assessing the correlation between hyponatremia or eunatremia in patients with acute stroke for severity and outcome. Int J Acad Med Pharm. 5, 218–221

[B5] Gankam Kengne F. (2023). Adaptation of the brain to hyponatremia and its clinical implications. J. Clin. Med. 12:1714. doi: 10.3390/jcm12051714 36902500 PMC10002753

[B6] Gankam Kengne F. Decaux G. (2017). Hyponatremia and the brain. Kidney Int. Rep. 3, 24–35. doi: 10.1016/j.ekir.2017.08.015 29340311 PMC5762960

[B7] Gray J. R. Morbitzer K. A. Liu-DeRyke X. Parker D. Jr Zimmerman L. H. Rhoney D. H. (2014). Hyponatremia in patients with spontaneous intracerebral hemorrhage. J Clin Med. 3, 1322–1332. doi: 10.3390/jcm3041322 26237605 PMC4470185

[B8] Ha C. Ryu J.-A. (2021). Association of hyponatremia and clinical prognosis in neuro critically ill patients. J. Neurointensive Care. 4, 30–35. doi: 10.32587/jnic.2021.00346

[B9] Hakim M. Mashfiqul-Hasan Islam M. Hossain M. A. Naznin J. Khan S. R. (2019). Frequency and types of hyponatremia in stroke patients admitted in a referral Neuroscience Institute of Dhaka. Clin. Neurol. Neurosci. 3, 46–49. doi: 10.11648/j.cnn.20190302.14

[B10] Higgins J. P. T. Thompson S. G. Deeks J. J. Altman D. G. (2003). Measuring inconsistency in meta-analyses. BMJ. 327, 557–560. doi: 10.1136/bmj.327.7414.557 12958120 PMC192859

[B11] Huang W-. Y. Weng W-. C. Peng T-. I. Chien Y.-Y. Wu C.-L. Lee M. . (2012). Association of hyponatremia in acute stroke stage with three-year mortality in patients with first-ever ischemic stroke. Cerebrovasc. Dis. 34, 55–62. doi: 10.1159/000338906 22759703

[B12] John B. P. S. Kumar A. Ashish Matthews S. Grewal S. S. (2025). Study of serum sodium levels in traumatic brain injury (TBI): an observational, cross-sectional, retrospective study. J. Contemp. Clin. Pract. 11, 533–540. doi: 10.61336/jccp/25-06-71

[B13] Kalita J. Singh R. K. Misra U. K. (2017). Cerebral salt wasting is the most common cause of hyponatremia in stroke. J. Stroke Cerebrovasc. Dis. 26, 1026–1032. doi: 10.1016/j.jstrokecerebrovasdis.2016.12.011 28110888

[B14] Khan A. Khan Z. Khan S. Ullah A. Ayub G. Tariq M. N. (2023). Frequency of hyponatremia and its impact on prognosis in ischemic stroke. Cureus. 15:e40317. doi: 10.7759/cureus.40317 37448406 PMC10337874

[B15] Kuramatsu J. B. Bobinger T. Volbers B. Staykov D. Lücking H. Kloska S. P. . (2014). Hyponatremia is an independent predictor of in-hospital mortality in spontaneous intracerebral hemorrhage. Stroke 45, 1285–1291. doi: 10.1161/STROKEAHA.113.004136 24713532

[B16] Liamis G. Barkas F. Megapanou E. Christopoulou E. Makri A. Makaritsis K. . (2019). Hyponatremia in acute stroke patients: pathophysiology, clinical significance, and management options. Eur. Neurol. 82, 32–40. doi: 10.1159/000504475 31722353

[B17] Malhotra A. Mittal P. Agrawal B. K. (2024). Serum electrolytes in acute stroke and their correlation with severity of stroke as well as short term clinical outcomes - a three month follow up study. Int. J. Med. Public Health. 14, 694–700. doi: 10.70034/ijmedph.2024.4.130

[B18] Moher D. Shamseer L. Clarke M. Ghersi D. Liberati A. Petticrew M. . (2015). Preferred reporting items for systematic review and meta-analysis protocols (PRISMA-P) 2015 statement. Syst. Rev. 4:1. doi: 10.1186/2046-4053-4-1 25554246 PMC4320440

[B19] Naidu M. A. Kumari K. A. (2025). Electrolyte imbalances in patients with acute stroke and effect of serum sodium levels on outcome of CVA: a retrospective study. Int. J. Acad. Med. Pharm. 7, 981–983. doi: 10.47009/jamp.2025.7.4.187

[B20] Padhy D. L. Panda D. R. Mohapatra D. D. Patro D. P. K. (2023). Hyponatremia as an independent indicator of grave prognosis in patients with spontaneous intracerebral haemorrhage. J. Cardiovasc. Dis. Res. 14, 1335–1341.

[B21] Pandya N. Patel K. L. Gandhi R. V. Solanki S. (2024). Prevalence and impact of hyponatremia in stroke patients: insights from a Tertiary Care Hospital, Gujarat, India. Eur. J. Cardiovasc. Med. 14, 674–679. doi: 10.61336/ejcm/24-4-92

[B22] Park S. J. Shin J. I. (2013). Inflammation and hyponatremia: an underrecognized condition? Korean J. Pediatr. 56, 519–522. doi: 10.3345/kjp.2013.56.12.519 24416046 PMC3885786

[B23] Podestà M. A. Faravelli I. Cucchiari D. Reggiani F. Oldani S. Fedeli C. . (2015). Neurological counterparts of hyponatremia: pathological mechanisms and clinical manifestations. Curr. Neurol. Neurosci. Rep. 15:18. doi: 10.1007/s11910-015-0536-2 25724319

[B24] Qian A. Zheng L. Duan J. Li L. Xing W. Tang S. (2025). Hyponatremia is associated with malignant brain edema after mechanical thrombectomy in acute ischemic stroke. BMC Neurol. 25:41. doi: 10.1186/s12883-025-04051-5 39875844 PMC11773710

[B25] Quinn L. Tian D. H. Fitzgerald E. Flower O. Andersen C. Hammond N. . (2020). The association between hyponatraemia and long-term functional outcome in patients with aneurysmal subarachnoid haemorrhage: a single centre prospective cohort study. J. Clin. Neurosci. 78, 353–359. doi: 10.1016/j.jocn.2020.06.003 32622650

[B26] Ranasinghe V. R. Bowatta G. Gawarammana I. B. (2023). Hyponatremia as an early marker of poor outcome of stroke - results of a prospective cohort study. Neurology Asia 28, 267–272. doi: 10.54029/2023pyf

[B27] Rodrigues B. Staff I. Fortunato G. McCullough L. D. (2014). Hyponatremia in the prognosis of acute ischemic stroke. J. Stroke Cerebrovasc. Dis. 23, 850–854. doi: 10.1016/j.jstrokecerebrovasdis.2013.07.011 23954607

[B28] Saadat P. Ahmadi Ahangar A. Haghshenas T. Alijanpour S. Rahmani A. (2022). Relationship between serum sodium level and impairment and disability in stroke patients. J. Babol Uni. Med. Sci. 24, 246–253.

[B29] Sah D. P. Mahaseth A. Sah P. (2024). Serum sodium in acute stroke and its clinical correlation. J. Natl. Med. College. 9, 45–50. doi: 10.3126/medphoenix.v9i2.73413

[B30] Salaudeen M. A. Bello N. Danraka R. N. Ammani M. L. (2024). Understanding the pathophysiology of ischemic stroke: the basis of current therapies and opportunity for new ones. Biomolecules. 14:305. doi: 10.3390/biom14030305 38540725 PMC10968326

[B31] Shah A. Sabir S. Artani M. Salam O. Khan S. Rizwan A. (2019). Significance of hyponatremia as an independent factor in predicting short-term mortality in patients with hemorrhagic stroke. Cureus 11:e4549. doi: 10.7759/cureus.4549 31275773 PMC6592831

[B32] Soiza R. L. Cumming K. Clark A. B. . (2015). Hyponatremia predicts mortality after stroke. Int. J. Stroke 10, 50–55. doi: 10.1111/ijs.12564 26178714

[B33] Yuen K. C. J. Sharf V. Smith E. Kim M. Yuen A. S. M. MacDonald P. R. (2022). Sodium and water perturbations in patients who had an acute stroke: clinical relevance and management strategies for the neurologist. Stroke Vasc. Neurol. 7, 258–266. doi: 10.1136/svn-2021-001230 34969834 PMC9240457

