# Supplementary material for: Association of hyponatremia and risk of in-hospital mortality in patients with acute stroke: a systematic review and meta-analysis
Source: Front Stroke. 2026 May 15;5:1816483. doi: 10.3389/fstro.2026.1816483 (PMC13218957; doi:10.3389/fstro.2026.1816483)
Supplement: Supplementary file 1 [file Table_1.docx]

***Table-S1****:* Summary of Search Results across Databases with Search Key Terms and results

| Databases | Search Key Terms | Search Results |
| --- | --- | --- |
| PubMed | ("Stroke"[Mesh] OR "Cerebrovascular Accident" OR "Cerebral Infarction" OR "Acute Stroke" OR "Brain Ischemia" OR "IntracerebralHemorrhage" OR "Hemorrhagic Stroke" OR "Ischemic Stroke")  AND  ("Electrolytes"[Mesh] OR "Serum Electrolytes" OR "Electrolyte Imbalance" OR "Hyponatremia" OR "Hypernatremia" OR "Hypokalemia" OR "Hyperkalemia" OR "Hypocalcemia" OR "Hypercalcemia" OR "Hypomagnesemia" OR "Hypermagnesemia" OR "Hypophosphatemia" OR "Hyperphosphatemia")  AND  ("Mortality"[Mesh] OR "Death" OR "In-Hospital Mortality" OR "30-day Mortality" OR "All-Cause Mortality")  AND  ("Risk Factors"[Mesh] OR "Risk Assessment" OR "Prognosis" OR "Outcomes" OR "Predictors" OR "Hazard Ratio" OR "Odds Ratio") | 127 |
| EMBASE | ('stroke'/exp OR 'cerebrovascular accident':ti,ab,kw OR 'cerebral infarction':ti,ab,kw OR 'acute stroke':ti,ab,kw OR 'brain ischemia':ti,ab,kw OR 'intracerebralhemorrhage':ti,ab,kw OR 'hemorrhagic stroke':ti,ab,kw OR 'ischemic stroke':ti,ab,kw)  AND  ('electrolyte blood level'/exp OR 'electrolyte imbalance':ti,ab,kw OR 'serum electrolytes':ti,ab,kw OR 'hyponatremia':ti,ab,kw OR 'hypernatremia':ti,ab,kw OR 'hypokalemia':ti,ab,kw OR 'hyperkalemia':ti,ab,kw OR 'hypocalcemia':ti,ab,kw OR 'hypercalcemia':ti,ab,kw OR 'hypomagnesemia':ti,ab,kw OR 'hypermagnesemia':ti,ab,kw OR 'hypophosphatemia':ti,ab,kw OR 'hyperphosphatemia':ti,ab,kw)  AND  ('mortality'/exp OR 'death':ti,ab,kw OR 'in-hospital mortality':ti,ab,kw OR '30-day mortality':ti,ab,kw OR 'all-cause mortality':ti,ab,kw)  AND  ('risk factor'/exp OR 'risk assessment':ti,ab,kw OR 'prognosis'/exp OR 'outcomes':ti,ab,kw OR 'predictors':ti,ab,kw OR 'hazard ratio':ti,ab,kw OR 'odds ratio':ti,ab,kw) | 842 |
| Scopus | TITLE-ABS-KEY("Stroke" OR "Cerebrovascular Accident" OR "Cerebral Infarction" OR "Acute Stroke" OR "Brain Ischemia" OR "IntracerebralHemorrhage" OR "Hemorrhagic Stroke" OR "Ischemic Stroke")  AND  TITLE-ABS-KEY("Serum Electrolytes" OR "Electrolyte Imbalance" OR "Hyponatremia" OR "Hypernatremia" OR "Hypokalemia" OR "Hyperkalemia" OR "Hypocalcemia" OR "Hypercalcemia" OR "Hypomagnesemia" OR "Hypermagnesemia" OR "Hypophosphatemia" OR "Hyperphosphatemia")  AND  TITLE-ABS-KEY("Mortality" OR "Death" OR "In-Hospital Mortality" OR "30-day Mortality" OR "All-Cause Mortality")  AND  TITLE-ABS-KEY("Risk Factors" OR "Risk Assessment" OR "Prognosis" OR "Outcomes" OR "Predictors" OR "Hazard Ratio" OR "Odds Ratio") | 1340 |

**Table-S2*:*** Summary of estimates based on subgroup analysis between hyponatremia and In-hospital mortality.

| **Variables** | **Subgroups items** | **No. of studies** | **RR (95% CI)** | **Degree of Heterogeneity** | |
| --- | --- | --- | --- | --- | --- |
|  |  |  |  | **I^2^ (%)** | **p values** |
| Stroke Subtype | IS | 4 | 1.29 (1.16, 1.42) | 0.0 | 0.921 |
|  | HS | 5 | 1.47 (0.70, 3.10) | 86.71 | 0.001 |
|  | IS+HS | 8 | 1.59 (1.19, 2.12) | 0.0 | 0.496 |
| Serum Sodium assessment time point | Within 24 hrs | 8 | 1.34 (0.96, 1.86) | 68.3 | 0.002 |
|  | Within 48 hrs | 3 | 1.33 (0.74, 2.41) | 45.3 | 0.161 |
|  | Within (24-72) hrs | 6 | 1.68 (1.00,2.81) | 59.9 | 0.020 |
| Ethnicity | Asian | 12 | 1.78 (1.34, 2.38) | 26.7 | 0.180 |
|  | Caucasian | 5 | 1.11 (0.84, 1.48) | 75.3 | 0.003 |
| **Overall** | | | 1.41 (1.14, 1.74) | 58.9 | 0.001 |
